# Selective cyclooxygenase-2 silencing mediated by engineered *E. coli* and RNA interference induces anti-tumour effects in human colon cancer cells

**DOI:** 10.1038/sj.bjc.6605859

**Published:** 2010-08-17

**Authors:** A Strillacci, C Griffoni, G Lazzarini, M C Valerii, S Di Molfetta, F Rizzello, M Campieri, M P Moyer, V Tomasi, E Spisni

**Affiliations:** 1 Department of Experimental Biology, University of Bologna, via Selmi 3, Bologna 40126, Italy; 2 Department of Clinical Medicine, St Orsola-Malpighi Hospital, via Massarenti 9, Bologna 40138, Italy; 3 INCELL Corporation, Cimarron Path 12734, San Antonio, TX 78249, USA

**Keywords:** COX-2, CRC, RNAi, *E. coli*

## Abstract

**Background:**

Cyclooxygenase-2 (COX-2) overexpression is strongly associated with colorectal tumourigenesis. It has been demonstrated that the chronic use of non-steroidal anti-inflammatory drugs (COX inhibitors) partially protects patients from colorectal cancer (CRC) development and progression but induces severe cardiovascular side effects. New strategies for selective COX-2 blockade are required.

**Methods:**

We developed an improved technique, based on RNA interference (RNAi), to gain a selective COX-2 silencing in CRC cells by a tumour-dependent expression of anti-COX-2 short-hairpin RNA (shCOX-2). Anti-COX-2 shRNA-expressing vectors were delivered in CRC cells (*in vitro*) and in colon tissues (*ex vivo*) using engineered *Escherichia coli* strains, capable of invading tumour cells (InvColi).

**Results:**

A highly tumour-dependent shCOX-2 expression and a significant COX-2 silencing were observed in CRC cells following InvColi strain infection. Cyclooxygenase-2 silencing was associated with a strong reduction in both proliferative and invasive behaviour of tumour cells. We also demonstrated a pivotal role of COX-2 overexpression for the survival of CRC cells after bacterial infection. Moreover, COX-2 silencing was achieved *ex vivo* by infecting colon tissue samples with InvColi strains, leading to anti-inflammatory and anti-tumour effects.

**Conclusion:**

Our RNAi/InvColi-mediated approach offers a promising tool for a highly selective COX-2 blockade *in vitro* and *in vivo*.

Colorectal cancer (CRC) represents the third most common tumour in humans and the second leading cause of tumour-related death in Western countries. In Europe, >4 00 000 new cases and >2 00 000 deaths per year are estimated (Ferlay *et al*, 2007). The mortality rate of CRC dramatically increases when tumour cells invade the primary tissue and generate lymph nodal and liver metastases. Owing to its high incidence and mortality rate, CRC is of great interest for the scientific community.

Over the last few decades, it has been demonstrated that the chronic use of non-steroidal anti-inflammatory drugs (NSAIDs), blocking isoforms 1 and 2 of the cyclooxygenase (COX) enzyme, confers a 40–50% reduction in the incidence of CRC (Phillips *et al*, 2002; Thun *et al*, 2002; Higuchi *et al*, 2003; Sandler *et al*, 2003). Cyclooxygenase is a key enzyme in the metabolism of arachidonate and regulates the synthesis of prostaglandins (H2, D2, E2, F2*α*, I2) and tromboxanes (TXA2 and TXB2), both having important roles as cellular mediators. Cyclooxygenase-2 represents the product of the *Cox-2* inducible gene. It is overexpressed in 40% of adenomas and in 80% of adenocarcinomas (Eberhart *et al*, 1994; Sano *et al*, 1995), and such an overexpression has been strongly associated with colon tumourigenesis both *in vitro* and in murine models (DuBois *et al*, 1996; Oshima *et al*, 1996; Williams *et al*, 1996). It is commonly accepted that COX-2 can contribute to CRC development and progression through mechanisms involving the stimulation of angiogenesis (Spisni and Tomasi, 1997; Iñiguez *et al*, 2003), the inhibition of apoptosis (Tsujii and DuBois, 1995) and the increase in cell migration/invasiveness (Yamauchi *et al*, 2002; Strillacci *et al*, 2006).

Celecoxib and rofecoxib were the first NSAIDs marketed as selective COX-2 inhibitors and they have been used in clinical trials to test their efficacy in the prevention and/or treatment of CRC. Unfortunately, celecoxib and, particularly, rofecoxib have been shown to significantly increase the risk of adverse cardiovascular events and myocardial infarction in treated patients (Bresalier *et al*, 2005; Solomon *et al*, 2005). Moreover, the cardiovascular toxicity of rofecoxib could be increased by its capability to directly inhibit prostacyclin synthase activity, as demonstrated by our group (Griffoni *et al*, 2007). Although celecoxib is still marketed, rofecoxib has been withdrawn from the worldwide market by Merck in September 2004.

RNA interference (RNAi) has rapidly become an innovative and elective tool for gene silencing at the post-transcriptional level. RNAi-mediated gene silencing can be achieved using small interfering RNA (siRNA) or short-hairpin RNA (shRNA) as precursor molecules. Although synthetic siRNAs are introduced in mammal cells using standard *in vitro* transfection methods, shRNAs are expressed after transfecting cells with plasmids (Lewis *et al*, 2002) or viral-based vectors (Brummelkamp *et al*, 2002). Numerous advantages come from shRNA-based RNAi approach: first, the possibility to obtain a long-term gene silencing; and second, the possibility to express shRNAs in a spatial- and/or temporal-specific manner using tissue-specific promoters or introducing regulatory elements on shRNA promoters.

In 2006, our research group demonstrated that it is possible to stably silence the COX-2 protein in a CRC cell line (HT-29) using the anti-COX-2 shRNA (shCOX-2). HT-29^pS(shCOX−2)^ showed a significant impairment of malignant behaviour (Strillacci *et al*, 2006) and a reduced ability to survive under hypoxic conditions (Sansone *et al*, 2009). To date, RNAi approaches have been used to efficiently silence the COX-2 protein in many different *in vitro* models (for a review, see Strillacci *et al*, 2010).

Several years ago, it was demonstrated that a functional gene transfer from bacteria to mammalian cells could occur. In particular, engineered *Escherichia coli*, expressing *Inv* and *HlyA* genes (from *Yersinia pseudotuberculosis* and *Lysteria monocytogenes*, respectively), was found to be able to invade and release DNA into host cells (Grillot-Courvalin *et al*, 1998). This phenomenon was also demonstrated *in vivo*, as it was observed that invasive *E. coli* can deliver therapeutic genes to the colonic mucosa in mice (Castagliuolo *et al*, 2005). Similarly, a successful transfer of shRNA into mammalian cells was achieved using non-pathogenic *E. coli* transformed with a plasmid containing expression cassettes for shRNA and *Inv*/*HlyA* genes. In 2006, Xiang *et al* (2006) applied this new strategy for the first time (termed ‘trans-kingdom RNAi’) to silence the *β*-1 catenin gene (*CTNNB1*) in human cells both *in vitro* and *in vivo*.

In this paper, we describe an effective method by which shCOX-2-expressing vectors can be delivered into CRC cells (*in vitro*) and in COX-2-overexpressing colonic tissues (*ex vivo*), using invasive *E. coli* strains to achieve a strong COX-2 silencing mediated by RNAi, coupled to anti-tumour effects. This strategy may prove to be suitable for an *in vivo* application aiming at COX-2 inhibition and CRC prevention.

## Materials and methods

### Cell lines

Human cancer cell lines HCA-7, HT-29, HCT-116, HeLa and human transformed kidney cell line HEK-293 were obtained from the American Type Culture Collection (Manassas, VA, USA). Normal human colon mucosal epithelial cell line NCM-460 (Moyer *et al*, 1996) was obtained after a licensing agreement with INCELL Corporation (San Antonio, TX, USA). Both HT-29^pS(−)^ and HT-29^pS(shCOX−2)^ cells have been previously created by our group, as described elsewhere (Strillacci *et al*, 2006). Cell lines were cultured at 37°C in 5% CO_2_ in Dulbecco's modified Eagle's medium (DMEM) supplemented with 10% heat-inactivated fetal calf serum (FCS), 2 mM L-glutamine and kanamycin 100 *μ*g ml^−1^. Both FCS and DMEM were purchased from Cambrex BioWhittaker (Charles City, IA, USA). The NCM-460 cells were cultured in the M3 base media obtained from INCELL Corporation. All other reagents were purchased from Sigma (St Louis, MO, USA).

### Colon tissue culture

Fresh colon biopsies were collected from patients affected by acute ulcerative colitis during endoscopy and immediately cultured in the MegaCell RPMI-1640 medium (Sigma). Patients gave written informed consent to participate in the study, which was approved by the local ethical committee.

### Plasmids

**Figure 1:**
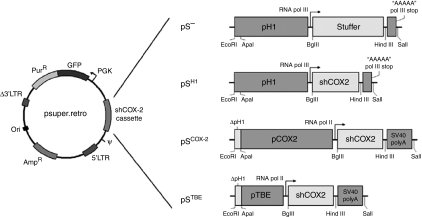
Scheme of pSUPER.retro vectors. pS^−^: original empty pSUPER.retro vector, with H1 promoter upstream ‘stuffer’ sequence; pS^H1^: pSUPER.retro vector in which shCOX-2 expression is controlled by H1 promoter; pS^COX−2^: pSUPER.retro vector in which shCOX-2 expression is controlled by human *Cox-2* promoter; pS^TBE^: pSUPER.retro vector in which shCOX-2 expression is controlled by a TBE-based promoter (carrying Tcf-binding elements).

The empty pSUPER.retro vector (pS^−^), based on the murine stem cell virus (MSCV) genome, was purchased from Oligoengine (Seattle, WA, USA). The pSUPER.retro vector expressing shCOX-2 under RNA pol III H1 promoter control (pS^H1^) was created as described elsewhere (Strillacci *et al*, 2006). pSUPER.retro vectors expressing shCOX-2 under control of human *Cox-2* promoter (pS^COX−2^) and TBE (Tcf-binding element)-based promoter (pS^TBE^) were created as follows: in both vectors, RNA pol III transcription stop signal was substituted with a SV40 polyA sequence between HindIII and SalI restriction sites (SV40 polyA sequence was amplified from the hER/pSG5 expression plasmid; forward primer: 5′-CCCAAGCTTAAATAAAGCAATAGCATCAC-3′ reverse primer: 5′-TAGAGTCGACCAGACATGATAAGAT-3′, 120 bp product); in pS^COX−2^, the H1 promoter (pH1) was substituted with the human *Cox-2* promoter sequence (pCOX-2) between ApaI and BglII restriction sites (pCOX-2 sequence was amplified from HCA-7 genomic DNA; forward primer: 5′-CGGGCCCTGAGCACTACCCATGATA-3′ reverse primer: 5′-GAAGATCTCCGAGAGAACCTTCC-3′, 1254 bp product); in pS^TBE^, pH1 was substituted with the TBE-based promoter sequence (pTBE) between ApaI and BglII restriction sites (the pTBE sequence was amplified from TOPFLASH plasmid, kindly provided by Dr Hans Clevers, University Hospital, Utrecht, The Netherlands; forward primer: 5′-CGGGCCCAAGCTATCAAAGGG-3′ reverse primer: 5′-CGAGATCTGGCGCCTCAGCTGGC-3′, 151 bp product). A comprehensive scheme of pS vectors described above is shown in Figure 1.

### Transfections

HCA-7, HT-29, HCT-116, NCM-460, HeLa and HEK-293 cells were seeded in 6-well plates (∼7 × 10^5^ cells per well) at 70% confluence. After 24 h, cells were transfected with pS^−^, pS^H1^, pS^COX−2^ and pS^TBE^ vectors using Lipofectamine 2000 transfection reagent (Invitrogen, Carlsbad, CA, USA) according to the manufacturer's instructions. After 6 h of incubation at 37°C, the transfection medium was replaced with 2 ml of complete medium containing 10% FCS. Cells were lysed 48 h after transfection for real-time PCR and western blot analyses.

### RNA extraction and real-time PCR

Total RNA from cultured cells was extracted using Eurozol reagent (Celbio, Milan, Italy) according to the manufacturer's instructions. Extracted RNA samples were treated with DNase I to remove any genomic DNA contamination using DNA-free kit (Ambion, Austin, TX, USA) and reverse transcribed using RevertAid First-Strand cDNA Synthesis Kit (Fermentas, Burlington, Ontario, Canada). Both COX-2 and *β*-glucuronidase (GUSB) mRNAs were reverse transcribed using random hexamer primers (Fermentas). The siCOX-2 was reverse transcribed using the stem-loop RT–PCR technique (Chen *et al*, 2005) using the following primer: 5′-GTCGTATCCAGTGCAGGGTCCGAGGTATTCGCACTGGATACGACAAATTCC-3′. U6 RNA was reverse transcribed using the following primer: 5′-AAAATATGGAACGCTTCACG-3′. Both COX-2 mRNA and siCOX-2 levels were analysed by real-time PCR using SYBR supermix (Bio-Rad, Hercules, CA, USA) and iCycler system (Bio-Rad) according to the manufacturer's instructions. The melting curve data were collected to check PCR specificity. Each cDNA sample was analysed in triplicate. Cyclooxygenase-2 mRNA levels were normalised against GUSB mRNA, whereas siCOX-2 expression was normalised against U6 RNA levels. Relative expressions were calculated using the formula 2^−2ΔCt^ values (ΔCt=Ct_gene_−Ct_hk_). Cyclooxygenase-2 primer pair: 5′-CCTGTGCCTGATGATTGC-3′ and 5′-CTGATGCGTGAAGTGCTG-3′ (165 bp product); siCOX-2 primer pair: 5′-GCAACTGCTCAACACCG-3′ and 5′-TGCAGGGTCCGAGGTAT-3′ (56 bp product); GUSB primer pair: 5′-TGGTATAAGAAGTATCAGAAGCC-3′ and 5′-GTATCTCTCTCGCAAAAGGAAC-3′ (297 bp product); U6 primer pair: 5′-CTTCGGCAGCACATATACT-3′ and 5′-AAAATATGGAACGCTTCACG-3′ (99 bp product).

### InvColi strains and bacterial infection

The *E. coli* (DH5*α* strain) was co-transformed with the pGB2-Ω-inv-hly plasmid (*Sm*^*R*+^, kindly provided by Dr Catherine Grillot-Courvalin, Institut Pasteur, Paris) and pSUPER.retro (*Amp*^*R*+^) vectors to obtain *E. coli* invasive strains carrying the three different shCOX-2 expression vectors (namely InvColi-pS^H1^, InvColi-pS^COX−2^ and InvColi-pS^TBE^) and the negative control (InvColi-pS^−^). Selection of co-transformed strains was based on both streptomycin and ampicillin resistance. Both HT-29 and HCA-7 cell lines were infected with InvColi strains according to the following procedure: cells were seeded in 25 cm^2^ flasks at 50% confluence (∼3 × 10^6^ cells) in an antibiotic-free medium (DMEM); InvColi strains were grown in LB medium at 37°C to reach an OD_600_ of ∼1.00; InvColi bacteria were then washed twice in fresh LB medium, re-suspended in antibiotic-free DMEM and added to cells’ media at an MOI (multiplicity of infection) of 1:1000; after 2 h of incubation at 37°C in 5% CO_2_, cells were washed and fresh DMEM medium supplemented with kanamycin was added to the flasks. Cells were lysed 48 h after infection for western blot and real-time PCR analyses. With regard to colon tissue samples, infection was achieved immediately after endoscopic resection by co-culturing biopsies and InvColi-pS strains in MegaCell RPMI-1640 medium as described above. Biopsies were lysed and supernatants were collected 48 h after infection for western blot, real-time PCR and Luminex (Luminex, Austin, TX, USA) analyses.

### Western blot

Cultured cells were homogenised in lysis buffer (50 mM Tris-HCl, pH 7.5, 2 mM EDTA, 100 mM NaCl, 1% Triton X-100, protease inhibitors mixture). Cell lysates were incubated 1 h on ice and centrifuged at 12 000 **g** to collect supernatants. After addition of SDS–PAGE sample buffer and boiling, 40 *μ*g of denatured proteins was separated in 12% SDS–PAGE and then transferred onto nitrocellulose papers. After blotting, nitrocellulose papers were incubated with specific antibodies. The primary antibodies used were polyclonal anti-COX-2 (Cayman Chemicals, Ann Arbor, MI, USA) and anti-*β*-actin (Sigma). Secondary antibodies (HRP conjugated) were purchased from Santa Cruz Biotechnology (Santa Cruz, CA, USA). Immunolabelling was visualised using the ECL procedure (Amersham Biosciences, Piscataway, NJ, USA). Bands were quantified using densitometric image analysis software (Quantity One, Bio-Rad). Normalisation was made against *β*-actin expression.

### Fluorescence and Inv-HlyA-pTBE sequence detection

InvColi pS^TBE^-infected cells were analysed 48 h after infection using the Nikon Eclipse 90i microscope (Nikon, Tokyo, Japan) to detect green fluorescent protein (GFP) expression. The total DNA of infected cells was extracted using phenol/chloroform procedure 24 h after infection, and the presence of pGB2-Ω-inv-hly and pS^TBE^ plasmids was confirmed by standard PCR technique, followed by agarose gel electrophoresis analysis. *Inv* primer pair: 5′-GCCAATAAGGAGCAGGAGAC-3′ and 5′-CCAAGGAGCCAGCCAATC-3′ (225 bp product); *HlyA* primer pair: 5′-CATTTCACATCGTCCATCTATTTG-3′ and 5′-TACCGTTCTCCACCATTCC-3′ (100 bp product); pTBE sequence primer pair (see above). The PCR analysis was also performed on *E. coli* co-transformed with pGB2-Ω-inv-hly and pS^TBE^ plasmids (pre-heated at 95°C for 5 min) and on purified plasmids themselves.

### Cell proliferation and viability analysis

Both HT-29 and HCA-7 cells were infected with InvColi strains. Twenty-four hours after infection (day 0), cells were seeded in 12-well plates (HT-29: 5 × 10^4^ cells per well; HCA-7: 2 × 10^4^ cells per well). On days 1, 2, 3, 6 and 8, cells were trypsinised, re-suspended in PBS 1 × and incubated for 10 min in 0.4% Trypan Blue (Sigma). Cells were then counted in a haemocytometer for unstained (viable) and stained cells. The percentage of cell viability was calculated as ((number of unstained cells)/(number of total cells)) × 100.

### Cell invasion assay

Invasion assay was performed using Boyden chambers (New Technologies Group, Milan, Italy) with 8-*μ*m pore polycarbonate membranes (New Technologies Group). Membranes were coated with Matrigel (Sigma) at 40-fold dilution. Assay was performed using fresh DMEM supplemented with 10% heat-inactivated FCS as the chemoattractant agent. Forty-eight hours after InvColi infection, viable HT-29 and HCA-7 cells were added into the upper chamber at high density (3 × 10^5^ cells) either in the absence or presence of phorbol 12-myristate 13-acetate (PMA) 40 nM stimulation and then incubated for 24 h at 37°C. After incubation, membranes were disassembled and non-invasive cells on the upper surface of the membrane were wiped with a cotton swab. The invasion index was determined by counting under light microscopy the number per optical fields ( × 200 magnification) of cells that migrated to the lower side of each membrane, after fixing and staining membranes with 2% Toluidine Blue.

### Cytokines, chemokines, growth factors and PGE_2_ detection

Simultaneous detection of 29 extracellular factors released by treated colon tissue samples was performed using Luminex technology based on multiplexed bead immunoassay. Before assay, cell media samples were concentrated 10 times using Microcon spin devices (YM3, Millipore, Billerica, MA, USA) and subsequently re-suspended in Bio-Plex (Bio-Rad) assay buffer. The levels of 27 cytokines, chemokines and growth factors (IL-1*β*, IL-1ra, IL-2, IL-4, IL-5, IL-6, IL-7, IL-8, IL-9, IL-10, IL-12(p70), IL-13, IL-15, IL-17, PDGF-bb, bFGF, G-CSF, GM-CSF, VEGF, TNF*α*, IFN*γ*, IP-10, Eotaxin, MCP-1, MIP-1*α*, MIP-1*β*, Rantes) were measured using the human ultrasensitive cytokine 27-plex antibody bead kit (Bio-Rad). The IL-1*α* level was measured using Milliplex Map kit (Millipore), whereas PGE_2_ levels were measured using the Luminex Prostaglandin E_2_ Kit (Cayman Chemicals). The assays were performed in 96-well filter plates, as per the manufacturers’ instructions. Concentration values were estimated using a standard curve and a fifth-order polynomial equation and were expressed as ng ml^−1^ or log (pg ml^−1^), after adjusting for the dilution factor (Bio-Plex Manager software 5.0, Bio-Plex). The intra-assay CV, including ultrafiltration and immunoassay, averaged 20%.

### Statistical analysis

Data were expressed as mean±s.e.m. of three independent experiments. Analysis of variance was used to assess the statistical significance of the differences. Differences were considered statistically significant at *P*<0.01.

## Results

### Selective expression of siCOX-2 in colon cancer cells

**Figure 2:**
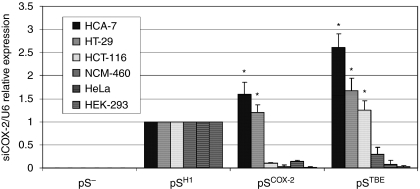
Selective expression of siCOX-2 in CRC cells. Six human cell lines were transfected with pS vectors, and siCOX-2 expression was evaluated by real-time PCR (48 h after transfection) and normalised against U6 RNA expression. For each cell line, relative expression of siCOX-2 refers to pS^H1^-transfected sample. Data represent mean±s.e.m. of three independent experiments. ^*^P<0.01.

In 2006, our group demonstrated that the COX-2 protein can be efficiently and stably downregulated by a constitutive expression of siRNA targeting COX-2 mRNA (siCOX-2) (Strillacci *et al*, 2006). The HT-29 colon cancer cell line was transduced with a retroviral vector (pSUPER.retro) containing an expressing cassette for shCOX-2 (siCOX-2 precursor molecule). In the pSUPER.retro vector, the transcription of shRNA sequence is driven by the human H1 promoter for RNA pol III (pS^H1^). H1-driven transcription leads to a constitutive expression of shRNA molecules in almost every different type of transduced human cells. To improve the selectivity of siCOX-2 expression, we modified the expression cassette of shCOX-2 by substituting the promoter element. In particular, we created the pS^COX−2^ vector, in which the H1 promoter (pH1) was substituted with the human *Cox-2* promoter (pCOX-2), and the pS^TBE^ vector, in which pH1 was substituted with a promoter containing TBEs (pTBEs), derived from TOPFLASH plasmid (largely used for luciferase assays). In both cases, shCOX-2 transcription is driven by RNA pol II that needs a polyA stop signal (which has been introduced in pS-modified vectors, downstream of shCOX-2 sequence) (Figure 1). Using this approach, we made shCOX-2 expression dependent on COX-2 and on Wnt/*β*-catenin cellular pathways, both highly activated in CRC cells. In fact, it is well known that CRC cells overexpress the COX-2 protein and this phenomenon depends on transcriptional (Yamamoto *et al*, 1995; Duque *et al*, 2005; Wu *et al*, 2005; Kaidi *et al*, 2006) and post-transcriptional regulation (Dixon *et al*, 2001; Strillacci *et al*, 2009). On the other hand, *β*-catenin is preferentially localised at the plasma membrane of normal colonocytes and promotes cellular adhesion, linking E-cadherin to the cytoskeleton. In CRC cells, as a consequence of mutations (Morin *et al*, 1997) and/or other cellular stimuli (Brabletz *et al*, 2001; Castellone *et al*, 2005), *β*-catenin frequently translocates in the nucleus and promotes the transcription of genes involved in cell proliferation, migration, survival, angiogenesis and stemness (Brabletz *et al*, 2005). In the nucleus, *β*-catenin associates with the transcriptional complex Tcf-4/Lef-1 (T-cell factor 4/lymphoid enhancer factor 1) and binds DNA on TBE promoter sequences. Figure 2 shows the expression levels of siCOX-2 in different human cell lines transiently transfected with pS^−^ (empty vector, negative control), pS^H1^, pS^COX−2^ and pS^TBE^ vectors. By transfecting CRC cell lines with pS^COX−2^ and pS^TBE^ vectors, it is possible to gain a highly selective expression of siCOX-2. In fact, in HT-29 and HCA-7 colon cancer cells, siCOX-2 expression driven by pCOX-2 and pTBE promoters resulted to be even higher than siCOX-2 expression driven by the pH1 promoter. As expected, in the HCT-116 colon cancer cell line (that does not express the COX-2 protein), we did not record a significant expression of siCOX-2 driven by pCOX-2, whereas siCOX-2 expression driven by pTBE was slightly higher than pH1. On the contrary, in NCM-460 (normal epithelial colon cells), HeLa (human cervix carcinoma cells) and HEK-293 (human transformed kidney cell line) cells, siCOX-2 expression driven by pCOX-2 and pTBE resulted to be very low, if compared with pH1-driven siCOX-2 expression.

### InvColi infection induces COX-2 silencing in CRC cell lines and significantly reduces PGE_2_ synthesis and release

**Figure 3:**
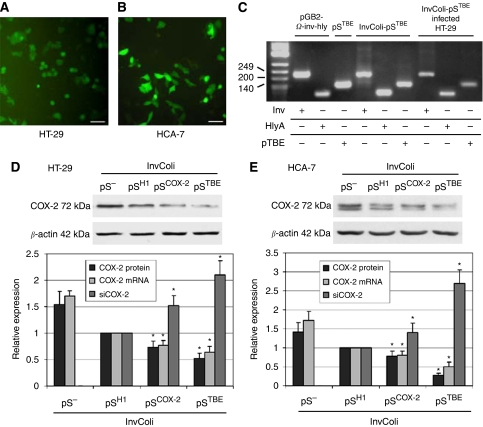
InvColi-pS strains infect CRC cells and induce high COX-2 silencing. GFP protein expression was evaluated in (**A**) HT-29 and in (**B**) HCA-7, 48 h after InvColi- pS^TBE^ infection (bar=30 *μ*m). (**C**) PCR analysis of Inv, HlyA and pTBE loci was performed on pGB2-Ω-inv-hly- and pS^TBE^-purified plasmids, on lysed InvColi-pS^TBE^ bacteria and on InvColi-pS^TBE^-infected HT-29 cells. PCR products were analysed after electrophoresis on 2% agarose gel. COX-2 protein, COX-2 mRNA and siCOX-2 levels were detected in (**D**) HT-29 and (**E**) HCA-7 cells, 48 h after InvColi strain infection, by western blot and real-time PCR. COX-2 protein, COX-2 mRNA and siCOX-2 expression was normalised against *β*-actin protein, GUSB (*β*-glucuronidase) mRNA and U6 RNA levels, respectively. Relative expression of COX-2 protein, COX-2 mRNA and siCOX-2 refers to InvColi-pS^H1^-infected sample. Data represent mean±s.e.m. of three independent experiments. ^*^P<0.01.

In 1998, Grillot-Courvalin *et al* (1998) showed that an engineered *E. coli* strain, expressing heterologous *Inv* and *HlyA* genes, was able to infect/invade mammalian cells and release DNA into host cells. On the basis of this approach, we developed a strategy to induce an RNAi-mediated COX-2 silencing in HT-29 and HCA-7 colon cancer cell lines. Bacterial LPS is known to induce COX-2 expression in human cells (Hla and Neilson, 1992). Thus, we first tested bacterial infection on CRC cells using InvColi-pS^−^ (see the ‘Materials and Methods’ section for details on InvColi-pS strains) at different MOIs. At an MOI value of 1:1000, we did not record any significant COX-2 overexpression due to the presence of bacterial LPS (data not shown). Second, we provided evidence that InvColi strains created in our laboratory were able to infect and invade human CRC cells at an MOI of 1:1000. To this purpose, we used the InvColi-pS^TBE^. pS^TBE^ vector, as the other pS vectors described above, carries a GFP reporter gene. Both HT-29 and HCA-7 cells infected with InvColi-pS^TBE^ started to express the GFP protein 48 h after infection (Figure 3A and B). An ∼90% infection/invasion efficiency was estimated on both cell lines. Moreover, soon after infection, it was possible to detect inside the cells, by PCR analysis, the presence of Inv-HlyA and pTBE sequences, carried by the pGB2-Ω-inv-hly plasmid and pS^TBE^ vector, respectively (Figure 3C).

**Figure 4:**
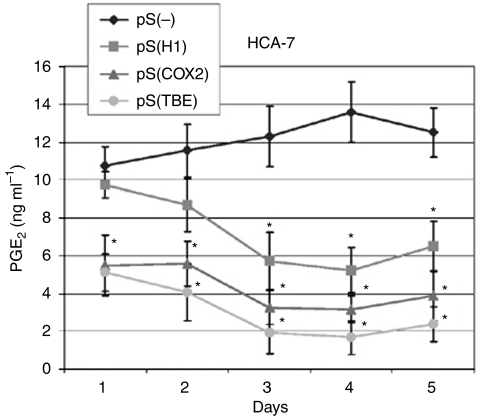
Effect of COX-2 silencing mediated by InvColi-pS on PGE_2_ production and release. Levels of PGE_2_ were quantified in conditioned media derived from InvColi-pS-infected HCA-7 cells, up to 5 days after infection. (*⧫*): cells infected with InvColi-pS^−^; (▪): cells infected with InvColi-pS^H1^; (▴): cells infected with InvColi-pS^COX−2^; (*•*): cells infected with InvColi-pS^TBE^. Analysis was carried out by a Luminex-based multiplexed bead immunoassay. Concentration values are expressed as ng ml^−1^ and represent mean±CV of three independent measurements, normalised considering the total protein amount extracted from each tissue sample. ^*^P<0.01.

Using the InvColi-mediated RNAi approach, we achieved a high COX-2 silencing in CRC cell lines. In fact, 48 h after infection with invasive InvColi strains transformed with shCOX-2-expressing vectors (InvColi-pS^H1^, InvColi-pS^COX−2^ and InvColi-pS^TBE^), we started to observe a significant reduction in COX-2 protein and mRNA levels in HT-29 and HCA-7 cells (Figure 3D and E). Interestingly, COX-2 silencing with InvColi-pS^TBE^ was more effective than the silencing obtained with InvColi-pS^−^ (negative control) or InvColi-pS^H1/COX−2^ bacteria. Most likely, the stronger COX-2 silencing obtained with InvColi-pS^TBE^ was dependent on a higher siCOX-2 expression, as detected in both HT-29 and HCA-7 cells (Figure 3D and E). Silencing results were highly comparable with those obtained after transfection of pS vectors into HT-29 and HCA-7 cells using the Lipofectamine 2000 Transfection Reagent (Invitrogen) (see Supplementary Material). We recorded a significant COX-2 protein silencing up to 5 days after InvColi-pS infection (data not shown). Very interestingly, prolonged COX-2 inhibition was associated with a strong impairment of PGE_2_ production (Figure 4) and, as previously demonstrated with western blot analysis, the strongest inhibitory effect was obtained after InvColi-pS^TBE^ infection.

We also evaluated whether COX-2 silencing induced by InvColi-RNAi might have been the result of an off-target effect depending on interferon system activation (in particular, the Jak-STAT pathway) (Sledz *et al*, 2003). Western blot analysis of phospho-STAT-1(Tyr701) (active form) levels, normalised against p85/p91 STAT-1 total protein levels, showed that the interferon system response was triggered only at high MOI values (1:100), whereas infection at MOI 1:1000 did not have any effect on STAT-1 phosphorylation (data not shown).

### COX-2 silencing mediated by RNAi after InvColi infection impairs proliferation, survival and invasiveness of CRC cells

**Figure 5:**
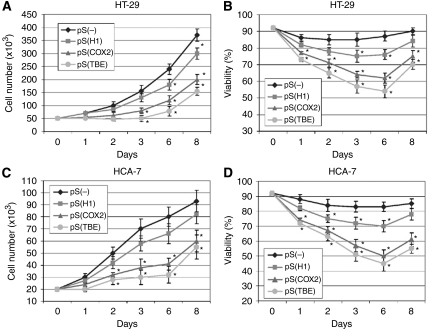
Effect of COX-2 silencing mediated by InvColi-pS on CRC cell proliferation and viability. Cell number and cell viability were evaluated in (**A**, **B**) infected HT-29 and (**C**, **D**) HCA-7 cells, respectively. (*⧫*): cells infected with InvColi-pS^−^; (▪): cells infected with InvColi-pS^H1^; (▴): cells infected with InvColi-pS^COX−2^; (*•*): cells infected with InvColi-pS^TBE^. Viability was evaluated using the Trypan Blue exclusion assay and quantified as ((number of unstained cells)/(number of total cells)) × 100. Data represent mean±s.e.m. of three independent experiments. ^*^P<0.01.

**Figure 6:**
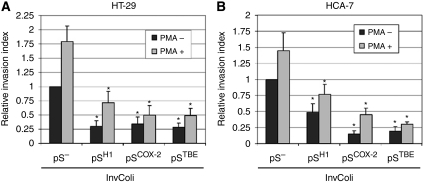
Effect of COX-2 silencing mediated by InvColi-pS on CRC cell invasiveness. Invasion index was evaluated in (**A**) HT-29 and (**B**) HCA-7 cells, 48 h after InvColi strain infection. The invasion assay was performed using Boyden chambers and 8-*μ*m polycarbonate membranes coated with Matrigel (40-fold dilution). Samples were tested in the absence (dark bars) and in the presence (light bars) of PMA 40 nM. Relative invasion index refers to HT-29 and HCA-7 cells infected with InvColi-pS^−^, not treated with PMA. Data represent mean±s.e.m. of three independent experiments. ^*^P<0.01.

As we have demonstrated an effective COX-2 silencing in CRC cells mediated by InvColi-pS strains, we have further investigated the phenotype of infected cells. Figure 5 shows data regarding proliferation and viability of COX-2-silenced HT-29 (Figure 5A and B) and HCA-7 (Figure 5C and D) cells and results were in high accordance in both cell lines. InvColi-pS^COX−2^ and, in particular, InvColi-pS^TBE^ infection induced a significant reduction in CRC cell proliferation and viability, despite InvColi-pS^H1^ infection that led only to a slight reduction. As COX-2 overexpression is involved in the malignant behaviour and invasiveness of CRC cells, we analysed the effect of the specific COX-2 silencing mediated by InvColi-RNAi in HT-29 and HCA-7 cells by performing a cell invasion assay, 48 h after InvColi infection. The invasion index of both cell lines was significantly reduced after COX-2 silencing (Figure 6A and B). Although in HT-29 we did not record any significant difference between the effectiveness of InvColi-pS^H1^, InvColi-pS^COX−2^ and InvColi-pS^TBE^, in HCA-7 InvColi-pS^COX−2^ and InvColi-pS^TBE^, infection induced a stronger reduction in the invasion index with respect to InvColi-pS^H1^. The impairment of the invasive behaviour of CRC cells due to RNAi-mediated COX-2 silencing was further confirmed after stimulation with PMA, which is known to induce COX-2 expression (Crofford *et al*, 1994) and promote cancer cell invasion (Han *et al*, 2000) (Figure 6A and B). These findings are in line with our previous work in which we demonstrated that a stable COX-2 downregulation in HT-29 cells, mediated by pH1-driven shCOX-2 expression, induces a significant impairment of malignant behaviour of HT-29 cells (Strillacci *et al*, 2006).

### CRC cell survival after bacterial infection depends on COX-2 overexpression

**Figure 7:**
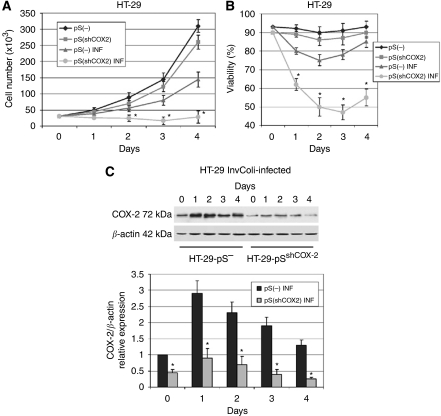
HT-29 cell survival after bacterial infection depends on COX-2 overexpression. (**A**) Cell number, (**B**) cell viability and (**C**) COX-2 protein expression were evaluated in HT-29^pS(−)^ and HT-29^pS(shCOX−2)^ cells infected with InvColi-pS^−^ (MOI 1:100), in comparison with control cells (not infected). See the ‘Materials and Methods’ section for details on cell lines. (*⧫*) HT-29^pS(−)^ cells, transduced with pS^(−)^ negative control vector; (▪) HT-29^pS(shCOX−2)^ cells, transduced with pS^(shCOX−2)^ vector and stably silenced for COX-2 protein; (▴): HT-29^pS(−)^ INF cells, transduced with pS^(−)^ negative control vector and infected with InvColi-pS^−^; (*•*): HT-29^pS(shCOX−2)^ INF cells, transduced with pS^(shCOX−2)^ vector and infected with InvColi-pS^−^. Viability was detected using the Trypan Blue exclusion assay and quantified as ((number of unstained cells)/(number of total cells)) × 100. COX-2 protein expression was evaluated by western blot and normalised against *β*-actin protein. COX-2 relative expression refers to not infected HT-29^pS(−)^ sample, day 0. Data represent mean±s.e.m. of three independent experiments. ^*^P<0.01.

**Figure 8:**
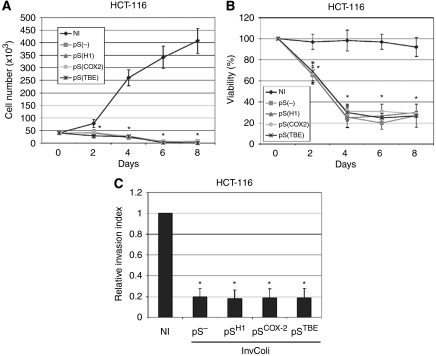
InvColi-pS infection impairs proliferation, viability and invasiveness of HCT-116 cells. (**A**) Cell number, (**B**) cell viability and (**C**) invasiveness were evaluated in HCT-116 cells infected by InvColi-pS strains (MOI 1:100). Viability was evaluated using the Trypan Blue exclusion assay and quantified as ((number of unstained cells)/(number of total cells)) × 100. (*⧫*): cells not infected, NI; (▪): cells infected with InvColi-pS^−^; (▴): cells infected with InvColi-pS^H1^; (*•*): cells infected with InvColi-pS^COX−2^; (^*^): cells infected with InvColi-pS^TBE^. The invasion assay was performed using Boyden chambers and 8-*μ*m polycarbonate membranes coated with Matrigel (40-fold dilution). Relative invasion index refers to HCT-116 cells not infected (NI). Data represent mean±s.e.m. of three independent experiments. ^*^P<0.01.

It is known that COX-2 has a key role in colon cancer progression and survival of colon cancer cells. Recently, we demonstrated that a COX-2/CA-IX (carbonic anhydrase IX) interplay controls CRC cell survival under hypoxic conditions (Sansone *et al*, 2009). Therefore, we aimed to demonstrate that COX-2 overexpression is also required for the survival of CRC cells after infection of invasive *E. coli* strains. As we have previously demonstrated (Strillacci *et al*, 2006), COX-2 stable silencing mediated by retroviral transduction did not affect proliferation and survival of HT-29 cells. In fact, we did not record any difference in the proliferation rate between HT-29^pS(−)^ (negative control) and HT-29^pS(shCOX−2)^ (in which COX-2 is stably downregulated). However, after a prolonged infection with invasive InvColi-pS^−^ (MOI 1:100, 6 h incubation, day 0), we observed a significant difference in cell proliferation and cell viability between HT-29^pS(−)^ and HT-29^pS(shCOX−2)^ cells (Figure 7A and B). At MOI 1:100, bacterial infection rapidly induced COX-2 overexpression in HT-29^pS(−)^ cells (days 1–4), most probably due to the higher presence of LPS (Hla and Neilson, 1992), but we did not observe this phenomenon in HT-29^pS(shCOX−2)^ cells (Figure 7C). The lack of high COX-2 protein levels in infected HT-29^pS(shCOX−2)^ cells led to a significant reduction in cell proliferation and viability. To further confirm the important role of the COX-2 protein in CRC cell survival, we performed InvColi-pS infection on HCT-116 cells, a CRC cell line not expressing the COX-2 protein. As expected, COX-2 protein and mRNA levels were not detectable in HCT-116 cells after 6 h infection with InvColi-pS strains at MOI 1:100 (data not shown). Importantly, the absence of COX-2 overexpression in infected HCT-116 cells led to a dramatic decrease in cell proliferation and viability (Figure 8A and B), as for HT-29^pS(shCOX−2)^ cells. Moreover, infected HCT-116 cells showed a reduced ability to invade Matrigel-coated filters (Figure 8C).

### COX-2 silencing mediated by InvColi strains in colon tissue samples

**Figure 9:**
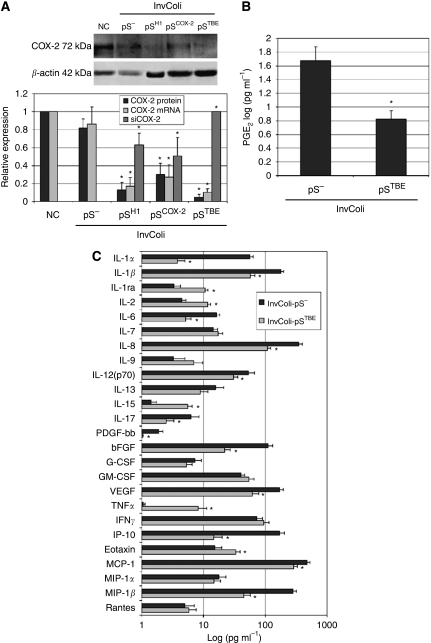
InvColi-pS strains infect colon tissue samples and induce high COX-2 silencing associated with anti-inflammatory and anti-angiogenic effects. (**A**) COX-2 protein, COX-2 mRNA and siCOX-2 levels were analysed in colon tissue samples by western blot and real-time PCR, 48 h after InvColi strain infection. COX-2 protein, COX-2 mRNA and siCOX-2 expression was normalised against *β*-actin protein, GUSB (*β*-glucuronidase) mRNA and U6 RNA levels, respectively. Relative expression of COX-2 protein and COX-2 mRNA refers to not infected samples (negative control, NC); relative expression of siCOX-2 refers to InvColi-pS^TBE^ infected samples. Data represent mean±s.e.m. of three independent experiments. ^*^P<0.01. (**B**) Levels of PGE_2_, (**C**) cytokines, chemokines and growth factors were quantified in InvColi-pS^−^- and InvColi-pS^TBE^-infected colon tissues conditioned media, 48 h after infection, by a Luminex-based multiplexed bead immunoassay. Concentration values are expressed as log(pg ml^−1^) and represent mean±CV of three independent measurements, normalised considering the total protein amount extracted from each tissue sample. ^*^P<0.01.

We evaluated the efficacy of COX-2 silencing *ex vivo* using InvColi-based RNAi on fresh colon tissue samples. To this purpose, samples were collected from patients affected by acute ulcerative colitis, supposed to be characterised by COX-2 protein expression. Levels of COX-2 protein, COX-2 mRNA and siCOX-2 were analysed 48 h after infection of biopsies with InvColi-pS strains (MOI 1:1000, 6 h incubation). As shown in Figure 9A, negative controls (untreated sample and sample infected with InvColi-pS^−^) were characterised by COX-2 expression, as expected. A significant COX-2 protein/mRNA reduction and siCOX-2 expression were recorded in samples infected with InvColi-pS^H1^, InvColi-pS^COX2^ and InvColi-pS^TBE^, compared with negative controls. Very importantly, InvColi-pS^TBE^ infection had the strongest effect, further confirming the *in vitro* data (see above). We also evaluated the effect of COX-2 silencing in colon tissues by analysing the levels of PGE_2_, cytokines, chemokines and growth factors present in the conditioned media 48 h after infection. For this analysis, only tissue samples infected with InvColi-pS^−^ (negative control) and InvColi-pS^TBE^ (which showed the most effective COX-2 silencing) were taken into consideration and all data collected are shown in Figure 9B and C. As expected, COX-2 protein silencing mediated by InvColi-pS^TBE^ led to a strong decrease in PGE_2_ secretion. Moreover, in media derived from InvColi-pS^TBE^-infected cells, we found a significant decrease in IL-1*α*, IL-1*β*, IL-6, IL-8, IL-12(p70), IL-17, PDGF-bb, bFGF, VEGF, IP-10, MCP-1 and MIP-1*β*, whereas we observed a marked increase in IL-1ra, IL-2, IL-15, TNF*α* and Eotaxin. Levels of IL-4, IL-5 and IL-10 were not detectable. Interestingly, COX-2 silencing led to a strong decrease in the secretion of pro-inflammatory cytokines such as IL-1*α*/*β* (Liu *et al*, 2003; Matsuo *et al*, 2009), IL-6 (Becker *et al*, 2005) or IL-8 (Brew *et al*, 2000), and that of pro-angiogenic growth factors such as PDGF-bb (Hsu *et al*, 1995), bFGF (Galzie *et al*, 1997) and VEGF (Takahashi *et al*, 1995). Furthermore, there was an increased secretion of some anti-inflammatory cytokines, such as IL-1ra (Konishi *et al*, 2005), and of cytokines with anti-tumour effect, such as IL-2 (Caporale *et al*, 2007) and IL-15 (Zhang *et al*, 2009). Similarly, TNF*α* increased secretion might exert an anti-tumour effect on colon cells, even if its role in CRC is still controversial (Koshiji *et al*, 1998; Popivanova *et al*, 2008). Thus, by a global analysis of *ex vivo* data, it results clear that InvColi-mediated RNAi induced COX-2 silencing and exerted an anti-inflammatory and anti-tumour effect on colon cells.

## Discussion

Over the last decades, a large body of experimental evidence has ascribed to the COX-2 enzyme an important role in colorectal tumourigenesis. Cyclooxygenase-2 is overexpressed in colon tumour tissues (Eberhart *et al*, 1994; Sano *et al*, 1995) and contributes to CRC progression and malignancy through mechanisms that act on cell proliferation/invasion (Yamauchi *et al*, 2002; Strillacci *et al*, 2006), apoptosis inhibition (Tsujii and DuBois, 1995) and tumour angiogenesis (Spisni and Tomasi, 1997; Iñiguez *et al*, 2003). It has been demonstrated that the chronic use of NSAIDs, inhibitors of COX activity, reduces CRC incidence in treated patients (Phillips *et al*, 2002; Thun *et al*, 2002; Higuchi *et al*, 2003; Sandler *et al*, 2003). Among these, ‘coxibs’ have been developed and marketed for the selective block of COX-2 activity. Celecoxib, rofecoxib, lumiracoxib and etoricoxib have been employed in many clinical trials to test their safety associated with the chronic use, but data collected are debated. The high incidence of cardiovascular adverse events is associated with the chronic use of coxibs (Bresalier *et al*, 2005; Solomon *et al*, 2005) and it seems clear that other pharmacological strategies, based on COX-2 inhibition, are required for the prevention and/or treatment of CRC.

Cyclooxygenase can be efficiently and selectively inhibited by RNAi techniques, either in a transient or a long-lasting manner (Strillacci *et al*, 2006). RNAi has become the first choice for gene silencing *in vitro* despite many disadvantages of RNAi approaches *in vivo*. RNAi-based silencing molecules can be delivered *in vivo* by intravenous injections or expressed by the use of viral-based systems. However, poor pharmacodynamics and/or heavy side effects reduce their use and effectiveness *in vivo*.

Hereby, we provide data supporting an alternative approach to target CRC cells and induce a highly selective COX-2 silencing, mediated by RNAi, both *in vitro* and *ex vivo*. In particular, we engineered invasive *E. coli* strains (InvColi) carrying siCOX-2 (siRNA sequences targeting COX-2 mRNA)-expressing vectors (pSUPER.retro, pS). These bacteria were able to infect/invade CRC cells and release their DNA contents, leading to a strong COX-2 silencing.

First, we modified the pS vector to gain a tumour-specific shCOX-2 expression. The basic pS vector allows a constitutive expression of shCOX-2 (siCOX-2 precursor) in almost every human cell line as shCOX-2 transcription is driven by the H1 promoter for RNA pol III (pS^H1^). Thus, we modified this expression cassette by substituting the H1 promoter with the human *Cox-2* promoter (in pS^COX−2^) or with a promoter sequence containing TBEs (in pS^TBE^), which represent the downstream target sequences on human genomic DNA of the Wnt/*β*-catenin-activated pathway. It is well known that *Cox-2* promoter activity is highly stimulated in CRC cells. Many cellular stimuli contribute to activate this phenomenon that leads to COX-2 overexpression (Yamamoto *et al*, 1995; Dixon *et al*, 2001; Duque *et al*, 2005; Wu *et al*, 2005; Kaidi *et al*, 2006; Strillacci *et al*, 2009). The Wnt/*β*-catenin pathway is highly activated in CRC cells and such an activation is caused by cellular stimuli or by mutation of proteins involved in the pathway, such as APC or *β*-catenin itself (Morin *et al*, 1997; Brabletz *et al*, 2001, 2005; Castellone *et al*, 2005). By the use of pS^COX−2^ and pS^TBE^ vectors, we demonstrated that siCOX-2 can be expressed in a more effective and selective manner in CRC cells. Under *Cox-2* or a TBE-based promoter control, siCOX-2 expression in CRC cell lines (HT-29 and HCA-7) was higher, if compared with H1-driven expression. On the contrary, siCOX-2 resulted in very low expression in normal colon epithelial cells (NCM-460) or in other human tumour cell lines (such as HeLa and HEK-293).

Second, we demonstrated that shCOX-2-expressing vectors (pS^H1^, pS^COX−2^ and pS^TBE^) can be efficiently delivered into HT-29 and HCA-7 CRC cell lines after infection with InvColi strains (MOI 1:1000), thus promoting a strong COX-2 downregulation (both at mRNA and protein levels) mediated by RNAi and a prolonged reduction in PGE_2_ levels. Most likely, this phenomenon led to the significant reduction in cell proliferation, viability and invasiveness observed in infected CRC cells. Very interestingly, shCOX-2 expression driven by the TBE promoter induced the highest COX-2 silencing, which resulted in a strong impairment of the tumour-associated phenotype. It seems clear that, by using the pS^TBE^ vector, it is possible to gain both efficacy and specificity for COX-2 silencing in CRC cells.

We used our model to further demonstrate the pivotal role of COX-2 overexpression in CRC cell survival. By infecting HT-29 cells stably silenced for COX-2 (HT-29^pS(shCOX−2)^, previously developed in our laboratory) with InvColi-pS^−^ at a higher MOI value (1:100), we observed a significant reduction in cell proliferation and survival, with respect to the negative control (HT-29^pS(−)^ cells). As it is well known that bacterial LPS induces COX-2 expression in human cells and, as we show herein, InvColi-pS^−^ infection at an MOI 1:100 induces COX-2 overexpression in CRC cells, our hypothesis is that the survival of CRC cells after bacterial infection could be strictly dependent on COX-2 induction. This observation was further confirmed in HCT-116 cells, a CRC cell line unable to express COX-2. In fact, the lack of COX-2 protein induction after InvColi-pS strain infection led to a significant impairment of cell proliferation, viability and invasiveness of HCT-116 cells. We did not record differences between the four InvColi-pS strains used for HCT-116 infection. InvColi-pS^−^ infection had the same effect of InvColi-pS^H1^, InvColi-pS^COX2^ and InvColi-pS^TBE^ infection. Thus, as for HT-29^pS(shCOX−2)^, the biological effect on HCT-116 cells was dependent on InvColi-pS infection *per se*, rather than on siCOX-2 expression. On the other hand, the global effect of InvColi-pS infection on CRC cell lines expressing COX-2 (HCA-7 and HT-29) is the result of COX-2 silencing mediated by RNAi, PGE_2_ levels reduction and cellular stress induced by bacteria infection.

Finally, in the light of a possible *in vivo* application, we tested *ex vivo* the efficacy of selective COX-2 silencing mediated by InvColi-based RNAi. Colon tissue samples, derived from patients affected by acute ulcerative colitis and expressing COX-2 protein, were infected with InvColi-pS strains and this resulted in a strong COX-2 inhibition at both mRNA and protein levels, especially after infection with InvColi-pS^TBE^. Cyclooxygenase-2 silencing was associated with a significant reduction in PGE_2_ and pro-inflammatory/pro-angiogenic factors (cytokines, growth factors) released by colon InvColi-pS^TBE^-infected cells. Moreover, we observed an increased production of factors with anti-inflammatory and anti-tumour effects.

In conclusion, we developed an RNAi-based strategy to efficiently and specifically silence the COX-2 protein in CRC cells, *in vitro* and *ex vivo*. Cyclooxygenase-2 downregulation, depending on InvColi-pS infection, induced a strong impairment of both proliferative and invasive behaviour of CRC cells *in vitro* and a significant anti-inflammatory and anti-tumour effect *ex vivo*. Taken together, our data demonstrate the feasibility of an RNAi-based/InvColi-driven approach for COX-2 silencing aimed at the prevention of CRC or the treatment of other inflammatory bowel diseases characterised by COX-2 overexpression. Using engineered *E. coli* strains or other non-pathogenic bacteria, siCOX-2 silencing could be locally delivered and expressed in colorectal tissues, thus promoting a blockade of COX-2 activity, supposedly deprived of systemic side effects.
