# Supplementary figures and images for: Selective cyclooxygenase-2 silencing mediated by engineered E. coli and RNA interference induces anti-tumour effects in human colon cancer cells
Source: Br J Cancer. 2010 Aug 17;103(7):975–86. doi: 10.1038/sj.bjc.6605859 (PMC2965869; doi:10.1038/sj.bjc.6605859)

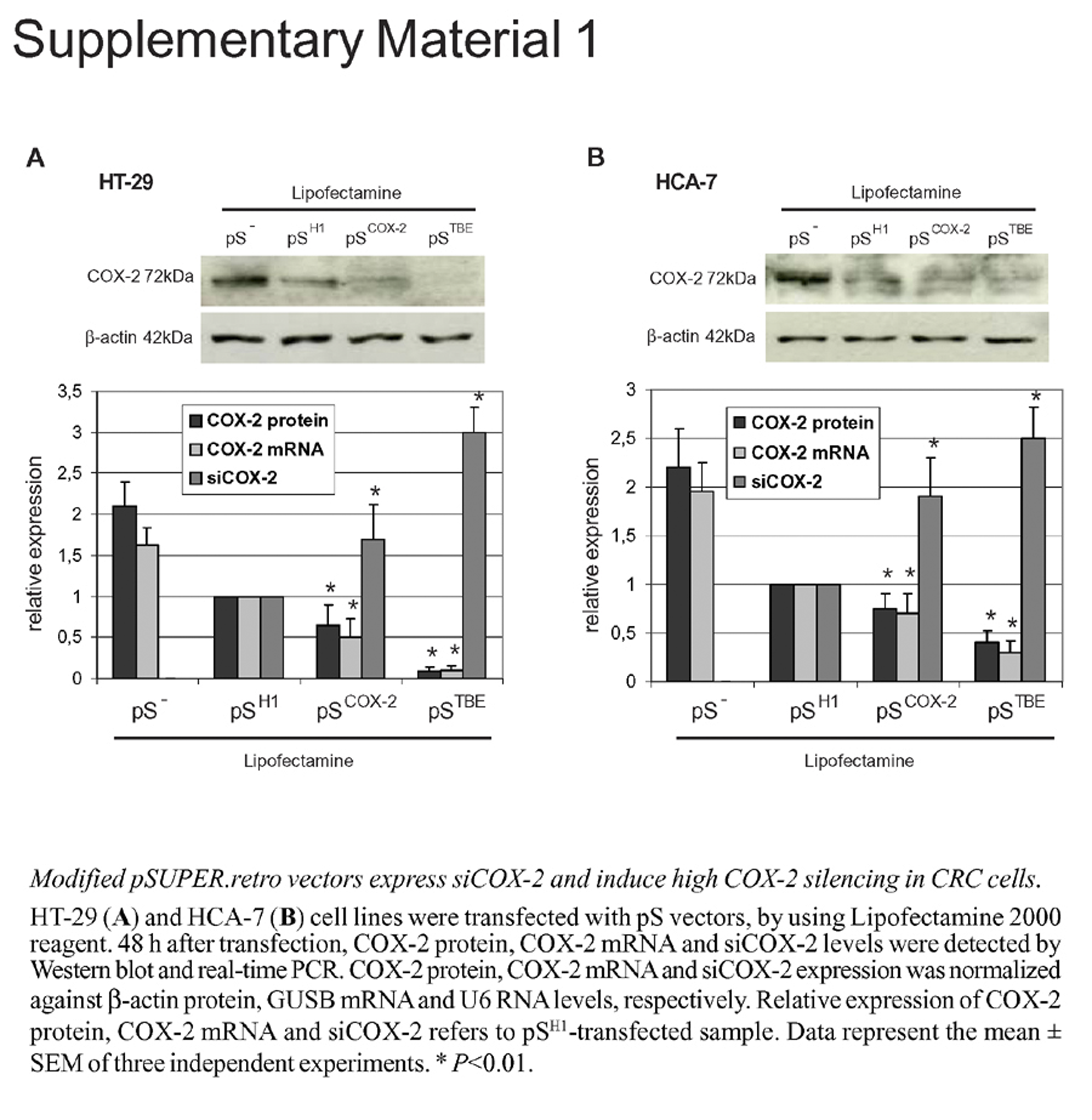

Supplement: Supplementary Material 1 [file 6605859x1.tif]
